# Pre**-**operative Endocrine Therapy

**DOI:** 10.1007/s12609-017-0255-6

**Published:** 2017-10-27

**Authors:** Laura M. Arthur, Arran K. Turnbull, Lucy R. Khan, J. Michael Dixon

**Affiliations:** 1 Edinburgh Breast Cancer Now Research Team, Division of Pathology Laboratories, Institute of Genetics and Molecular Medicine, Western General Hospital, The University of Edinburgh, Crewe Road South, Edinburgh, EH4 2XU Scotland; 2 Edinburgh Breast Unit, Western General Hospital, Crewe Road South, Edinburgh, EH4 2XU Scotland

**Keywords:** Breast cancer, Neoadjuvant, Endocrine therapy, Aromatase inhibitor, Pre-operative, Tamoxifen

## Abstract

**Purpose of Review:**

Pre-operative endocrine therapy can be used to down-stage large or locally advanced breast cancers in ER+ disease. In the last four decades, it has evolved from a treatment perceived as an alternative to surgery for those too unfit to undergo surgery or chemotherapy, to the present day where it is a valuable and valid option in the treatment of postmenopausal women with ER-rich (Allred score 7–8, or > 50% staining for ER) breast cancer.

**Recent Findings:**

Emerging data from the metastatic setting is translating into neoadjuvant trials, utilising dual endocrine targeting or combinations of endocrine agents and other targeted drugs, including those acting against components of the PI3K pathway and the cell cycle. The routine use of peri-operative endocrine therapy in all ER+ tumours may help to yield important long-term prognostic information, and guide adjuvant endocrine therapy.

**Summary:**

Pre-operative endocrine therapy is an exciting and evolving area with emerging new approaches. In this review, established evidence and emerging data on its applications are discussed.

## Introduction

Approximately 75% of all invasive breast cancers express oestrogen receptor alpha (ER+), a favourable prognostic factor and a strong predictor of response to endocrine therapies. In early breast cancer, treatment usually includes surgery, followed by adjuvant (post-operative) treatments, involving one or more of chemotherapy, endocrine therapy, radiotherapy and anti-HER2-targeted therapies in appropriate patients. Neoadjuvant therapy refers to treatment given prior to surgery and can be used to down-stage large or locally advanced breast tumours, making initially unresectable disease become operable [1] and increasing the likelihood of successful breast conservation surgery [2]. Neoadjuvant chemotherapy became established as an option for many women with breast cancer following studies in the 1970s and 1980s. There is now increasing evidence for the role of neoadjuvant endocrine therapy as an alternative in ER+ breast cancers, especially in postmenopausal women [3]. This review will focus on the evidence available for neoadjuvant endocrine therapies in ER+ breast cancer.

## Application of Neoadjuvant Therapy in Breast Cancer

Neoadjuvant therapy allows patients with large operable and locally advanced breast cancer who respond well to treatment to be suitable for breast conservation surgery at a later date. A further advantage is the unique in vivo observation of tumour response to treatment [4]. This can identify patients who do not respond to a particular drug, allowing a switch to an alternative or combination approach, or indeed proceeding to surgery rather than continuing with neoadjuvant systemic therapy. Ultimately, this has potential to spare patients from unnecessary and ineffective treatments. It also allows a unique opportunity to explore potential biomarkers associated with response or resistance through sequential biopsies taken on treatment in the neoadjuvant period. Longer-term prognostic information can also be derived in this early period. Response in the neoadjuvant setting is reported by varying methods between trials, including clinical response (measured by clinical calliper examination, or amenability to breast conservation surgery as assessed by a breast surgeon), radiological response (tumour volume as assessed by imaging, including mammography, ultrasound or magnetic resonance imaging (MRI)) or pathological response (assessed on sequential biopsy by reduction in Ki67). Pathological complete response (pCR, an absence of invasive and in situ disease after treatment) is associated with favourable prognosis with improved long-term disease-free and overall survival; especially in HER2-positive and triple-negative breast cancer subtypes [5], however, it is attained less frequently with neoadjuvant endocrine therapy than with chemotherapy.

## Indications for Neoadjuvant Endocrine Therapy

Patients who may be candidates for neoadjuvant treatment include those with large operable primary cancers greater than 5-cm diameter (T3), or any cancer that is not immediately amenable to breast conservation surgery due to its size, cancers with skin or chest wall involvement (T4), or those with involved axillary lymph nodes at diagnosis (N1–2) [6]. There are also circumstances where women with smaller primary tumours may be considered for neoadjuvant therapy, such as if they wish to undergo breast conservation surgery rather than mastectomy and/or have a small breast to tumour size ratio [7].

In premenopausal women in whom neoadjuvant therapy is deemed necessary, chemotherapy rather than endocrine therapy is currently recommended. If chemotherapy is not an option due to patient preference or comorbidities, premenopausal patients should proceed to surgery rather than receiving neoadjuvant endocrine therapy. There have been studies of neoadjuvant endocrine therapy in premenopausal women, and it appears that it is effective in shrinking cancers; however, the sample numbers in published studies are too small and are lacking in long-term clinical outcome data, to be certain that this is a safe option [8]. In postmenopausal women with locally advanced breast cancer, neoadjuvant chemotherapy is widely used, but in women with ER-rich HER2-negative disease, neoadjuvant endocrine therapy is an appropriate alternative. Judicious review with serial examination and imaging to assess response to neoadjuvant endocrine therapy is required in these patients, and an alternative approach (a switch in endocrine agent or surgery) is required if there is suggestion of disease progression. A meta-analysis comparing neoadjuvant endocrine therapy with neoadjuvant chemotherapy in 20 randomised controlled trials of 3490 women found similar response rates with neoadjuvant endocrine therapy and neoadjuvant chemotherapy, but lower toxicities associated with endocrine therapy. Neoadjuvant endocrine monotherapy with an aromatase inhibitor compared with neoadjuvant combination chemotherapy achieved similar clinical response rate (odds ratio (OR) 1.08, 95% CI 0.5–2.35, *p* = 0.85), radiological response rate (OR 1.38, 95% CI 0.92–2.07, *p* = 0.12) and breast conservation surgery rate (OR 0.65, 95% CI 0.41–1.03, *p* = 0.07) but with lower toxicity [9]. Most studies included in this meta-analysis did not directly compare endocrine therapy with chemotherapy, but rather two endocrine agents or an endocrine agent with or without growth factor pathway inhibitors.

Attainment of pCR after neoadjuvant therapy correlates to improved event-free and overall survival [10]. ER+ (luminal) tumours have a more favourable prognosis overall than other more proliferative breast cancer subtypes. However, the rate of pCR achieved with neoadjuvant chemotherapy is less in luminal disease than that in triple-negative or HER2-positive disease. In one study of 107 patients treated with neoadjuvant anthracycline-based chemotherapy, only 7% of luminal disease attained pCR compared to 36% of HER2+/ER− (< 5% ER staining) tumours and 27% of triple negative tumours (*p* = 0.01) [11]. The degree of response to neoadjuvant chemotherapy is also inversely related to the level of ER expression, with a significantly higher rate of pCR in patients with ER- and PR-negative disease (17.7%), compared with those with low expression (0–49% staining) of ER and PR (3.3%), and those with high expression (> 50% staining) of ER and PR (0%; odds ratio 14.4, *p* < 0.001) [12]. Indeed, the survival advantage offered by adjuvant chemotherapy in luminal A breast cancer (ER-rich, HER2-negative, low proliferative) is doubtful. The International Breast Cancer Study Group IX trial found no survival advantage of cyclophosphamide, methotrexate and 5-fluorouracil followed by tamoxifen, over tamoxifen alone, in 1669 postmenopausal women with ER+ node-negative breast cancer at 13.1 years follow-up (64 vs 66%, *p* = 0.99) [13]. Therefore, this cohort of patients may be able to safely avoid chemotherapy and its associated toxicities [14], and these women may be candidates for neoadjuvant endocrine therapy, if neoadjuvant therapy is deemed necessary.

The rate of pCR in response to neoadjuvant endocrine therapy in postmenopausal women with ER+ disease is not significantly superior to the rate of pCR with chemotherapy in ER+ disease (3 vs 6% in one study of 239 patients [8] and < 10% overall in the 20 study meta-analysis [9]). However, the rate of breast conservation surgery is improved (33 vs 24%, *p* = 0.058) [8]. This highlights the limitation of using pCR after neoadjuvant therapy to predict outcome in ER+ disease. The better outcomes in this cohort are largely due to the benefit of adjuvant endocrine therapy. The use of tamoxifen as a neoadjuvant agent was first described in the 1970s as an alternative to surgery in elderly patients with comorbidities [15], where it was used as primary endocrine therapy as an alternative to surgery, rather than a neoadjuvant approach. A meta-analysis of 7 trials and 1571 elderly patients found no overall survival advantage of surgery over primary endocrine therapy in this cohort (hazard ratio (HR) 0.98, *p* = 0.9). It did show however a benefit in progression-free survival in favour of surgery followed by adjuvant endocrine therapy over primary endocrine therapy alone (HR 0.55, *p* = 0.0006) [16]. Therefore, traditionally neoadjuvant endocrine therapy was reserved for patients deemed too frail to undergo surgery or receive chemotherapy. However, the undisputed benefit of endocrine therapy in the adjuvant setting in ER+ disease has driven the exploration and increasing use of endocrine agents in the neoadjuvant setting. The increasing use of the aromatase inhibitors (AIs), and their superior tolerability over chemotherapy, has proved an effective and safe option as neoadjuvant therapy followed by surgery, for select postmenopausal women [17].

Tumours that are most likely to respond to neoadjuvant endocrine therapy are ER-rich (Allred score 7–8, or > 50% staining for ER), with a low proliferative index (Ki67 < 10–15%). Response also correlates to level of ER expression. In one study of 324 postmenopausal women, response to neoadjuvant letrozole was more than 60%, and to tamoxifen 45%, in patients with Allred scores of 7–8, compared to 0% in Allred scores 0–2 [18]. Postmenopausal women with ER-rich, HER2-negative primary breast cancers may be treated with neoadjuvant endocrine therapy. In elderly and frail patients, neoadjuvant endocrine treatment may down-size a primary breast cancer to allow a less extensive resection under local anaesthesia in the future [19]. In patients who are deemed so frail that they are unlikely to ever be considered as surgical candidates, regardless of response to neoadjuvant therapy, or whose life expectancy is likely to be limited by an illness other than breast cancer, up-front primary endocrine therapy is a reasonable approach [20] and can be continued for months to years. The same agent can be used long term so long as disease is responsive or stable, and a switch to an alternative agent can be considered if there is evidence of disease progression, which is likely eventually in the absence of definitive local therapy.

## Neoadjuvant Endocrine Therapies; Tamoxifen or Aromatase Inhibitors?

### Postmenopausal Women

The third-generation aromatase inhibitors (AIs) letrozole, anastrozole and exemestane have become established in the care of ER+ breast cancer in the adjuvant setting due to their superiority over tamoxifen in postmenopausal patients with improved disease-free and overall survival [21, 22]. This has translated to the neoadjuvant setting too, with several trials demonstrating superiority of AIs over tamoxifen.

The P024 trial confirmed superiority of 4 months of neoadjuvant letrozole over tamoxifen in 337 patients with early breast cancer who were initially not eligible for breast conservation surgery. Overall objective response rate, determined as partial or complete response as assessed by clinical palpation, was 55% with letrozole and 36% with tamoxifen (*p* < 0.001). Rate of breast conservation surgery was also superior with letrozole (45 vs 35%, *p* = 0.022) [23]. Letrozole also correlated with a greater reduction in mean Ki67 (87% in letrozole, 75% with tamoxifen) [24]. In ER+HER2+ disease, the superiority of letrozole over tamoxifen was even more significant, with overall response rates of 88 vs 21%, *p* = 0.0004 [18].

The Immediate Preoperative Anastrozole, Tamoxifen, or Combined with Tamoxifen (IMPACT) trial compared neoadjuvant tamoxifen, anastrozole or both for 3 months, in 330 women with ER+ invasive, non-metastatic operable, or locally advanced but potentially operable breast cancer. There was no significant difference in objective response rates between the groups, but there was an increased rate of breast conservation in the anastrozole group over tamoxifen group (46 vs 22%, *p* = 0.03) [25].

In the Preoperative Anastrozole Compared with Tamoxifen (PROACT) trial, 12 weeks of neoadjuvant anastrozole and tamoxifen yielded similar response rates by calliper (50 vs 46.2%; OR 1.24, 95% CI 0.84–1.83, *p* = 0.29) and ultrasound measures (39.5 vs 35.4%, respectively; OR 1.19, 95% CI 0.82–1.72, *p* = 0.37) in 451 patients with ER+ large operable or potentially operable breast cancer. This trial included patients receiving neoadjuvant chemotherapy at the same time; and when excluding these, improved response was seen with anastrozole over tamoxifen (ultrasound response 36.2 vs 26.5%, OR 1.57 (0.97–2.55) *p* = 0.07; calliper response 49.7 vs 39.4%, OR 1.5 (0.96–2.34) *p* = 0.08). Additionally, surgical options were improved. Patients with tumours deemed inoperable at baseline had improved response with anastrozole over tamoxifen (ultrasound response 52 vs 29%, OR 1.81 (1.06–3.11) *p* = 0.03; calliper response 48.6 vs 35.8%, OR 1.69 (1.03–2.78) *p* = 0.04) [26].

A Russian study comparing 3 months of neoadjuvant exemestane to tamoxifen in 151 patients with T2N1–2, T3N0–1 or T4N0M0 disease confirmed superiority with exemestane, with improved clinical response rate (76.3 vs 40%, *p* = 0.05) and increased breast conservation rate (36.8 vs 20%, *p* = 0.05) [27].

A meta-analysis of these four trials incorporating 1160 patients confirmed superiority of neoadjuvant AIs over tamoxifen in postmenopausal women. Response was superior when assessed by clinical objective response rate (RR 1.29 (1.14–1.47) *p* < 0.001), ultrasound response rate (RR 1.29 (1.10–1.51) *p* = 0.002) and breast conserving surgery rate (RR 1.36 (1.16–1.59) *p* < 0.001) [28]. Toxicities and tolerability were similar for all drugs. Similarly, in a separate meta-analysis comparing seven trials of neoadjuvant AIs with tamoxifen, AIs were associated with a significantly higher clinical response rate (OR 1.69 (1.36–2.10) *p* < 0.001, *n* = 1352), radiological response rate (OR 1.49 (1.18–1.89) *p* < 0.001, *n* = 1418) and breast conservation surgery (BCS) rate (OR 1.62,(1.24–2.12) *p* < 0.001, *n* = 918) compared with tamoxifen [9].

### Premenopausal Women

There is limited evidence on the role of neoadjuvant endocrine therapy in premenopausal women. A study assessing 7 days of pre-operative tamoxifen in 44 patients (58% postmenopausal, 32% pre- or perimenopausal) found a mean decrease in Ki67 of 40% (95% CI 29–63%) [29]. The STAGE trial compared 24 weeks of neoadjuvant anastrozole with tamoxifen in 197 premenopausal women receiving goserelin (gonadotropin-releasing hormone agonist, GnRH). They demonstrated superiority of anastrozole over tamoxifen in attaining complete or partial response as measured clinically with callipers (70.4 vs 50.5%, *p* = 0.004), ultrasound (58.2 vs 42.4%, *p* = 0.027) and MRI or computed tomography (CT) (64.3 vs 37.4%, *p* = 0.032). More patients also achieved breast-conserving surgery with anastrozole over tamoxifen (86 vs 68%) [30]. Long-term outcomes from both studies are not yet available however.

Many premenopausal breast cancer patients will have adverse prognostic features, and in general, neoadjuvant chemotherapy rather than endocrine therapy in this group is preferred. The Grupo Español de Investigación del Cáncer de Mama (GEICAM) study compared neoadjuvant chemotherapy with 4 cycles of epirubicin and cyclophosphamide followed by 4 cycles of docetaxel, with neoadjuvant endocrine therapy with 24 weeks of exemestane, in 95 patients with luminal breast cancer. The cohort included 51 premenopausal patients, who were randomised to endocrine therapy (exemestane combined with goserelin) or chemotherapy. Clinical response rate was significantly superior with chemotherapy compared to endocrine therapy in premenopausal patients (75 vs 44%, *p* = 0.027) [31]; however, the rate of BCS was not significantly different (47% with chemotherapy, 56% with endocrine therapy, *p* = 0.24). Overall, considering the poorer response compared to neoadjuvant chemotherapy, there is no established role for neoadjuvant endocrine therapy in premenopausal women.

## Choice of Aromatase Inhibitor

The American College of Surgeons Oncology Group (ACOSOG) Z1031 trial compared the three third-generation AIs for 16–18 weeks prior to surgery in 377 postmenopausal women with ER-rich (Allred 6–8) T2–4, N0–3, M0 breast cancers. They found no significant differences between anastrozole-, letrozole- and exemestane-treated groups with objective response rates of 69.1, 74.8 and 62.9%, respectively, and breast conservation rates of 64, 42.1 and 48.1%, respectively. Rates of down-staging to allow breast conservation were improved with all AIs with no one drug being superior [32]. Similar rates of down regulation of ER, PR and reduced Ki67 have also been shown with short course (14 days) of anastrozole and letrozole pre-operatively, with no significant difference between these drugs [33].

## Duration of Treatment

Most trials of neoadjuvant endocrine therapy have treated patients for a duration of between 3 and 6 months. Prolonging neoadjuvant letrozole beyond this has also been shown to increase response rates. One study of 182 women treated with neoadjuvant letrozole described clinical and ultrasound response after 3 months in 69.8%, which improved to 83.5% in a cohort of 62 patients who continued letrozole for longer than 3 months. Tumour volume continued to reduce between 3 and 6 months (median 50%), between 6 and 12 months (median 37%) and between 12 and 24 months (median 33%). Rate of breast conservation increased from 60 to 72% in those who continued treatment for longer than 3 months [34].

A further study of 116 ER+ women treated with neoadjuvant exemestane found an increase in objective response rate with ultrasound and callipers from 47.4% at 16 weeks to 50.9% at 24 weeks. Breast conservation surgery as assessed by a breast surgeon, was deemed possible in 49.1% at diagnosis and 76.7% after 24 weeks of neoadjuvant exemestane [35].

Furthermore, a prospective multicentre trial of 146 patients treated with neoadjuvant letrozole found that the median time taken to attain breast conservation surgery was 7.5 months (95% CI 6.3–8.5 months) [36]. This supports the theory that longer treatment may provide progressive tumour shrinkage and may be required to achieve breast conservation surgery. It is important to closely monitor these patients throughout neoadjuvant treatment and switch to surgery or alternative systemic therapy should there be sign of disease progression.

## Neoadjuvant Chemotherapy vs Neoadjuvant Endocrine Therapy

Comparisons of neoadjuvant chemotherapy and endocrine therapy are limited reflecting the fact that neoadjuvant chemotherapy is less effective in ER+ disease than ER− in achieving pathological complete response (8% in ER+ cancers compared to 24% of ER−, *p* < 0.001) [37]. One trial compared neoadjuvant endocrine therapy (3 months of exemestane or anastrozole) with neoadjuvant chemotherapy (4 cycles of doxorubicin and paclitaxel) in 239 postmenopausal women with ER+ breast cancer. There was no significant difference in clinical response in endocrine-treated vs chemotherapy-treated groups (64.5 vs 63.6%) or in mammographic response (60 vs 63%). There was however a trend in favour of increased breast conservation in the endocrine-treated rather than chemotherapy-treated groups (33 vs 24%, *p* = 0.058) [8].

The GEICAM study compared neoadjuvant endocrine therapy with chemotherapy in 95 women and demonstrated superiority of chemotherapy in premenopausal women, as mentioned above. This was not reflected across the whole cohort however, with clinical response of 66% with chemotherapy vs 48% with endocrine therapy (*p* = 0.075), and was even less marked in postmenopausal women, 57% chemotherapy vs 52% endocrine therapy, *p* = 0.78 [31].

The Neoadjuvant Endocrine vs Chemotherapy Trial (NEOCENT) was designed to compare neoadjuvant chemotherapy with endocrine therapy in postmenopausal women with ER-rich (Allred 6–8) tumours. Eighty patients were randomised to receive 6 cycles of 5-fluorouracil, epirubicin, and cyclophosphamide or 18–23 weeks of letrozole. They described similar rates of clinical and radiological response between the two cohorts; however, the trial closed early due to slow recruitment and is underpowered; therefore, no significant conclusions can be drawn [38]. There are few other prospective trials directly comparing the effects of neoadjuvant endocrine therapy with chemotherapy [9].

## Prediction of Treatment Benefit

The neoadjuvant period provides a unique opportunity to re-biopsy a tumour on treatment, allowing the exploration of biomarkers predictive of response and resistance to treatment. The proliferation antigen Ki67 is an accepted prognostic factor at baseline [39]. The level of Ki67 after a period of neoadjuvant treatment [40] is thought to offer a reliable prognostic estimate. It can correlate with long-term outcome after as little as 2 weeks of neoadjuvant treatment, as demonstrated in the IMPACT trial [41]. The limited use of pCR in luminal breast cancers to neoadjuvant therapy has led to exploration of other biomarkers, including Ki67, to predict response to treatment and estimate prognosis.

One study assessed 21-gene recurrence score (RS; Oncotype DX) at diagnostic core biopsy in 43 patients treated with neoadjuvant tamoxifen or anastrozole. In those with a low-risk RS (< 18), response was 64%, compared with 31% each for both intermediate (19–30) and high RS (> 31). There was a non-significant trend to improved progression free survival at 5 years in the low-RS group, compared with intermediate and high (100 vs 84%, 73%, respectively, *p* = 0.14), although this study is limited by its small cohort [42]. A further study assessed OncotypeDx in 116 patients receiving 24 weeks of neoadjuvant exemestane. Clinical response was significantly higher in patients with a low RS compared with high RS (59.4 vs 20% *p* = 0.015). Rate of breast conservation was also significantly better in low RS vs high RS (90.6 vs 46.7%) [43]. Of note, ER-related genes are heavily weighted in calculation of the recurrence score.

The Preoperative Endocrine Prognostic Index (PEPI) was developed from the 228 patient cohort of the P024 trial and independently validated in the 203 patients in the IMPACT cohort. PEPI is calculated from tumour size, nodal stage, ER and Ki67 of the surgical specimen following neoadjuvant endocrine treatment, giving a score of 0 (good prognosis), 1–3 (intermediate) and 4+ (poor prognosis) which correlated to recurrence-free survival (log rank *p* = 0.002) [44]. PEPI 0 patients with early-stage tumours had no recurrence events in 5 years in the training cohort, highlighting an excellent prognostic group who could forego adjuvant chemotherapy. The currently recruiting ALTERNATE trial will prospectively assess validity of PEPI in response to anastrozole, fulvestrant or both.

A 4-gene signature, able to predict response to neoadjuvant AI based on expression of two genes pretreatment, namely IL6ST (associated with immune signalling) and NGFRAP1 (apoptosis induction related), and two proliferation genes (ASPM and MCM4) after 2 weeks of letrozole, has been developed. This model has a 96% accuracy (96% sensitivity, 94% specificity; positive predictive value 98%, negative predictive value 89%) [45]. Blinded independent validation in a second cohort treated with neoadjuvant anastrozole yielded similar results, predicting response correctly in 40 of 44 patients; 91% accuracy, 90% sensitivity and 92% specificity. The 4-gene signature also correlates with progression-free and breast cancer-specific survival in the training cohort. Measures of the 4 genes by qRT-PCR and their proteins by IHC have also been validated, markedly increasing the reproducibility of this test.

The ACOSOG Z1031B study comparing neoadjuvant letrozole, anastrozole and exemestane assessed an on-treatment biopsy after 2–4 weeks. If Ki67 was > 10%, patients were deemed to be non-responsive to AI and switched to neoadjuvant chemotherapy or proceeded to surgery. Forty nine of 236 (20.7%) women had Ki67 > 10% at 2 weeks, of these 35 switched to chemotherapy, and pathological complete response was achieved in two patients. At 4.4 years follow-up, they also had a significantly increased risk of relapsed disease (log rank *p* = 0.004) [46]. Gene expression assessed by MultiGene Proliferation Score (MGPS) RNA assay demonstrated even in those with Ki67 > 10% at 2 weeks, the non-responders, there was still down regulation of MGPS, although to a lesser degree than in those with Ki67 < 10%. One limitation of this study is the use of pCR to assess response to chemotherapy in ER+ tumours, which may explain the low rate demonstrated. Further survival data is also awaited.

These studies confirm the added value of an on-treatment biopsy in predicting response to neoadjuvant endocrine therapy, and indeed long-term outcome. There are several other ongoing trials looking to prospectively explore this further, using on-treatment biopsy assessment of response to alter decisions about ongoing treatment.

The trial of Peri-Operative Endocrine Therapy: Individualising Care (POETIC) is a phase III randomised prospective multicentre trial which recruited 4486 patients over 5 years, comparing 4-week peri-operative AI (2 weeks pre- and 2 weeks post-operative) with no therapy. The primary endpoint is to assess outcome in the peri-operative AI group, over surgery followed by standard adjuvant therapy group. A secondary outcome is to determine the most effective time points for molecular profiling, comparing measures of Ki67 at baseline and at 2 weeks, in prediction of recurrence-free and overall survival [47]. Recruitment closed in 2014 and patients are being followed up annually for 10 years.

## Combination Therapies

Studies in the metastatic setting have proven the benefit of combinations of endocrine agents with other endocrine drugs, or targeted agents, including CDK4/6 inhibitors, and drugs targeting the PI3K pathway, such as mTOR inhibitors and pan PI3K inhibitors. These findings are now being translated into trials of these approaches in the neoadjuvant setting also.

Palbociclib, a CDK4/6 inhibitor, was approved for use in metastatic ER+ breast cancer by the US Food and Drug Administration in 2015 following the PALOMA-1 trial, where in combination with letrozole as first-line therapy in 165 women, it improved progression-free survival from 10.2 to 20.2 months over letrozole alone (*p* = 0.0004) [48]. In a larger multicentre cohort in PALOMA-2, there was also improved progression-free survival with letrozole and palbociclib over letrozole and placebo as initial treatment in metastatic ER+ disease (24.8 vs 14.5 months, *p* < 0.001, *n* = 666) [49]. The PALTAN study is a phase II neoadjuvant trial of palbociclib in combination with letrozole and trastuzumab in early ER+HER2+ breast cancer, which commenced June 2017 (*NCT02907918*). The toxicities of CDK4/6 inhibitors can be problematic however, with neutropenia experienced by 75% of patients in PALOMA-1 and leukopenia in 43%, which may result in dose reductions and interruptions. Febrile neutropenia is much less common however (< 1.8%) [48, 49]. One aim of PALTAN is to assess the tolerability and safety of palbociclib in combination with letrozole and trastuzumab in the neoadjuvant setting.

The NeoPalAna study assessed neoadjuvant palbociclib combined with anastrozole in 50 patients with ER+HER2− disease to assess complete cell cycle arrest (CCCA), defined as Ki67 < 2.7% after 2 weeks of treatment with both drugs. CCCA rate was significantly higher after adding palbociclib to anastrozole (87 vs 26%, *p* < 0.001) [50]; however, there was a rebound effect in Ki67 after discontinuation of palbociclib, suggesting a maintenance treatment may be required. Neutropenia was experienced in 56% of patients, requiring seven (3.5%) to have dose reductions.

The NeoMONARCH phase II randomised multicentre study of 224 patients compared abemaciclib (CDK4/6 inhibitor) alone, abemaciclib plus anastrozole, or anastrozole alone for 2 weeks pre-operatively. Ki67 assessed at baseline and again at 2-week biopsy was significantly more suppressed at a 9-month interim analysis with abemaciclib either as monotherapy or in combination (*p* < 0.001, *n* = 64) than with anastrozole alone [51]. Again, we await with interest survival outcomes in these patients.

One study compared 4 months of neoadjuvant letrozole plus placebo with letrozole plus everolimus (mTOR inhibitor) in 270 postmenopausal ER+ patients. Clinical response was superior in the everolimus arm (68.1 vs 59.1%, *p* = 0.62), as was a reduction in Ki67 to less than 1%, 57% in everolimus arm compared with 30% of placebo arm, *p* < 0.01 [52]. The toxicity of everolimus may limit its adoption into routine use in the neoadjuvant setting.

The LORELEI trial is currently ongoing to assess the PI3K inhibitor, taselisib, combined with letrozole in the neoadjuvant period.

The ALTERNATE trial is a further prospective trial currently recruiting to compare neoadjuvant anastrozole or fulvestrant or combination of both, for 6 months in T2–3, N0–3, M0 ER+HER2− disease. After 4 weeks, Ki67 will be assessed, and patients switched to neoadjuvant chemotherapy if > 10%. Patients will continue the same endocrine drug for adjuvant therapy and PEPI will also be assessed. The primary objective is to determine whether endocrine resistance (progressive disease clinically or radiologically) is less with fulvestrant, or fulvestrant plus anastrozole, than anastrozole alone. Secondary aims will examine degree of Ki67 suppression amongst others [53].

## Conclusion

Neoadjuvant endocrine therapy is a suitable treatment for postmenopausal women with ER-rich, HER2-negative, low-grade, low proliferative breast cancers. AIs are more effective than tamoxifen. Duration of treatment should be at least 4 months, but can be extended if necessary to achieve breast conservation, so long as the patient is monitored for any sign of disease progression. Repeat biopsy and assessment of on-treatment Ki67 during neoadjuvant treatment can provide valuable prognostic information. It is likely in the future that there will be an increased use of pre- and peri-operative endocrine therapy in ER+ disease, as is being explored currently in POETIC. This could provide valuable prognostic information and help to guide adjuvant endocrine therapies.
